# Hypnotism in America

**Published:** 1892-03-19

**Authors:** 


### The Doctor's Armchair.

### HYPNOTISM IN AMERICA.

They do some things better in America than we do. They are about to try, at any rate in the State of New York, to put hypnotisers and hypnotism under rational control. A Bill has already been introduced into the Legislature, which, if passed into law, will at least put an end to some of the discreditable scandals which disgrace the name of science. One of the provisions of the Bill is that "It shall be unlawful for any person, not a duly licensed physician, to hypnotise another." A further provision is that "It shall be unlawful for any person, except a duly licensed physician in the course of lectures to medical students, or before scientific bodies, to give exhibition of, or perform, hypnotic demonstrations in public." It is also provided that "Any person violating either of the foregoing provisions shall be guilty of a misdemeanour."

The right way to form a correct judgment on proposals like the foregoing is to appproach the subject from a historical and practical, and not from an *a priori* point of view. In this country sober people have been grievously scandalised by various hypnotic displays, and self-respecting medical men have been disgusted. In America, so far as we can learn, matters have been infinitely worse. There seem to be more fools in America than we have here; and there are certainly many more knaves. Hypnotism in the great Republic has been the pastime of the trifler, a fortune to the cheat, and a refuge for the destitute and unsuccessful medical practitioners. There seems to be prevalent in the expansive West a kind of mental inflation which lends itself with infinite readiness to the arts of the trickster and the charlatan. The average American is so awake to the wonderful that there is nothing which he cannot believe. *Credo quia impossible* is a mere trifle compared with his promptitude in the swallowing of wonders. Hence it is that not only Hypnotism, but Mormonism, Prophet Harrisism, and every other conceivable and inconceivable absurdity under the sun has found a home among the mixed nationalities which constitute the people of America.

In this country we are more sober. But even we, with all our insularity and love of prescriptive propriety, have not escaped the contagion of modern necromancies. It is to be sorrowfully admitted that in England also foolish persons may here and there be found, if one does but search with sufficient candle-power; and even knaves are not altogether unknown to the police and other experts. We are bound to confess that the medical profession itself has not altogether avoided the taint of the prevailing epidemic. Some British doctors have gravely taken up hypnotism as a method of therapeusis, and have pocketed guineas with a beautiful benevolence as if they were conscious of having given to the patient more than his *quid pro quo*. If the mischief had begun and ended with doctors no great harm would have been done, because the profession, as a whole, has never believed in hypnotism, and, therefore, it would have taken prompt measures to stamp out a knavish heresy if an impulsive public had not insisted that there must be some fire when the smoke of credence was so intense and universal.

At last, however, the public itself has seen the bladder pricked and all the wind let out. It is difficult to find a man or a woman, even in a fashionable West-end drawing-room or a first-class railway carriage, who believes any longer in what so short a time ago was the most fascinating fad of the times. As for the medical profession, they are resolved to have done with the business, and all the more promptly and sternly because for a brief period they lent an almost willing ear to what they knew in their hearts was foolish and false.

But, it may be asked, if hypnotism be so foolish and false, why not abolish it altogether by statute? Why permit even the members of the medical profession to practise so unsatisfactory an art? To which it may be replied that, if the members of the medical profession are alone permitted to practise hypnotism, the thing will speedily die out of itself. The mere name connotes practically nothing at all. Certain persons have nerve power whereby they can exercise a certain amount of control over other persons. It may sometimes be desirable, in the interests of patients, that this kind of nervous control be exercised over them. When it is desirable, the medical man who possesses sufficient nerve force will exercise the control; and there will be an end to the business. The name by which the medical hypnotiser calls his work is of no importance whatever. It may be one of half-a-dozen — "Hypnotism," "Mesmerism," "Animal Magnetism," "Neurism," anything one likes that bears any reasonable approximation to the indefinable something which may have been attempted and done.

The thing which some of us still call hypnotism should certainly be placed in the sole charge of the members of the medical profession, because they alone are fitted by training, knowledge, and character to use it wisely and well. If the general public are left to act as they please in the matter, what is, and will continue to be, the inevitable result? The intelligent and the conscientious, being aware that they do not understand anything at all about it, carefully leave the subject alone. But the unscrupulous and the idle, looking about in search of an easy living, find in hypnotism a ready means of deceiving an ignorant public, and of gaining both notoriety and pelf at the same time. Is this the sort of thing that should be encouraged by the state in a scientific, but highly nervous age? The educated intelligence of the public should refuse to tolerate it; and those who perceive the increasing neurosity of whole classes of the population should insist upon making their voices heard in defence of their less instructed fellow citizens. We congratulate the members of the New York Legislature in that they have resolved to deal sharply with a demonstrated evil. It is to be hoped that some of the more intelligent members of one of our own Houses of Parliment will set about ensuring the same protection for the people of this country. The question is one which the House of Lords might very well deal with, and we commend it to the consideration of young and public-spirited Peers.

